# Proteomic Analysis Reveals Autophagy as Pro-Survival Pathway Elicited by Long-Term Exposure with 5-Azacitidine in High-Risk Myelodysplasia

**DOI:** 10.3389/fphar.2017.00204

**Published:** 2017-04-26

**Authors:** Alessandra Romano, Cesarina Giallongo, Piera La Cava, Nunziatina L. Parrinello, Antonella Chiechi, Calogero Vetro, Daniele Tibullo, Francesco Di Raimondo, Lance A. Liotta, Virginia Espina, Giuseppe A. Palumbo

**Affiliations:** ^1^Divisione di Ematologia, Azienda Ospedaliera Policlinico UniversitariaCatania, Italy; ^2^Scuola Superiore di CataniaCatania, Italy; ^3^Center for Applied Proteomics and Molecular Medicine, George Mason UniversityManassas, VA, USA

**Keywords:** myelodysplastic syndrome, azacytidine, autophagy

## Abstract

Azacytidine (5-AZA) is the standard first-choice treatment for high-risk myelodysplasia (MDS) patients. However, the clinical outcome for those patients who interrupt treatment or whose disease failed to respond is very poor. In order to identify the cellular pathways that are modified by long-term exposure to 5-AZA, we evaluated key proteins associated with the autophagy pathway by reverse-phase microarray (RPPA). Comparing bone marrow mononucleated cells (BMMCs) obtained from 20 newly-diagnosed patients and after four 5-AZA cycles we found an increased autophagy signaling. We then evaluated *ex-vivo* the effect of the combination of 5-AZA with autophagy inhibitors chloroquine (CQ) and leupeptin. Since 5-AZA and CQ showed synergism due to an increase of basal autophagy after 5-AZA exposure, we adopted a sequential treatment treating BMMCs with 5 μM 5-AZA for 72 h followed by 10 μM CQ for 24 h and found increased apoptosis, associated to a reduction of G2M phase and increase in G0-G1 phase. Long-term exposure to 5-AZA induced the reduction of the autophagic marker SQSTM1/p62, reversible by CQ or leupeptin exposure. In conclusion, we identified autophagy as a compensatory pathway occurring in MDS-BM after long-term exposure to 5-AZA and we provided evidences that a sequential treatment of 5-AZA followed by CQ could improve 5-AZA efficacy, providing novel insight for tailored therapy in MDS patients progressing after 5-AZA therapy.

## Introduction

Myelodysplastic Syndromes (MDS) are a heterogeneous group of bone marrow (BM) diseases characterized by peripheral cytopenias due to ineffective hematopoiesis and susceptibility to leukemic transformation. MDS are characterized by an uncoupling of proliferation and differentiation at the level of the hematopoietic stem cells (HSCs). According to the International Prognostic Scoring System (IPSS), MDS are distinguished into low- and high-risk subtypes based on the number of hematopoietic deficits, the percentage of marrow blasts, and cytogenetic pattern (Greenberg et al., 1997; Sanz et al., 1998; Valent et al., 2007; Della Porta et al., 2015). Approximately one third of patients present with high-risk disease (Int-2 and High IPSS scores), associated with a significant probability of leukemia transformation with a corresponding lower apoptotic index and higher percentage of marrow blasts.

In IPSS high-risk MDS patients, several therapeutic approaches have been used to kill abnormal blasts, including intensive conventional chemotherapy and low-dose cytarabine (Valent et al., 2007; Greenberg, 2011). Patients can sometimes achieve long-term remission or cure with allogeneic hematopoietic stem cell transplantation, but only those who are healthy enough to undergo the procedure can benefit from this type of therapy.

Azacytidine (5-AZA) is a pyrimidine nucleoside analog and a DNA methyltransferase inhibitor, thus resulting in a reduction of DNA methylation and altered gene expression that might, in turn, help to restore normal hematopoiesis (Raj et al., 2007). Actually, 5-AZA is the first choice of therapy for high-risk MDS patients for low toxicity, achievement of hematological response and increased survival times (Fenaux et al., 2009; Silverman et al., 2011). However, the outcome for MDS patients who progressed after an initial clinical response is poor (Barresi et al., 2010), with a median overall survival was of 5.6 months (Prebet et al., 2011).

Little is known about pro-survival compensatory pathways induced by chronic exposure to 5-AZA, to address the clinical question about the best therapeutic option in patients previously-treated with 5-AZA and to personalize the treatment at first relapse.

We hypothesized that during chronic, long-term exposure to 5-AZA pro-survival pathways could be elicited in malignant clones, thus to emerge at the treatment interruption. Although the small size of the MDS clone in the BM hinders deep mechanistic biomolecular investigations, to test our hypothesis we performed a proteomic characterization of a relatively large panel of primary, patient-derived BM mononuclear cells (BMMCs) before and after 5-AZA long-term exposure.

Our study revealed autophagy as a pro-survival pathway in MDS-BMMCs elicited by long-term treatment with 5-AZA, that could be triggered in those patients who failed to respond after initial clinical benefit.

## Methods

### Patients

Between January 2009 and July 2010, 40 samples of bone marrow mononuclear cells (BMMCs) were collected from 20 patients, affected by newly-diagnosed high-risk MDS (defined in accord to IPSS criteria), before and after treatment with 5-AZA, using density gradient centrifugation (Ficoll-Hypaque, GE Healthcare, Roosendal, The Netherlands) and saved in our bio-bank at −80°C.

Baseline characteristics of patients and response after 4 or 8 cycles are reported in Table 1. Treatment consisted of 8 cycles of 75 mg/m^2^/day for 7 days + 21 days of wash-out. Sample collection was performed before start treatment (Time 0) and after 4 cycles (after 112 ± 12 days from baseline Time 1), achieving at least a hematological response, to focus the study to early responder patients.

**Table 1:** **Characteristics of patients evaluated for RPPA**.

**Characteristics at diagnosis**	
Male/Female (n)	13/7
Median age (range)	71 (59–81)
Median Hemoglobin, g/dL (range)	7.8 (6.8–10.2)
Median Platelet count,10^3^/mmc (range)	56 (50–89)
Median White Blood Cell count,10^3^/mmc (range)	2.7 (1.9–4.2)
Normal /Complex karyotype/Failed cytogenetics	13 /4/3
IPSS INT-2/HIGH (n)	14/6
**Response after 4 cycles**	
Complete remission	0
Partial remission	2
Hematological improvement	14
Stable disease	4
Failure	0
**Response after 8 cycles**	
Complete remission	1
Partial remission	2
Hematological improvement	7
Stable disease	5
Failure	2
Cytogenetic response	3

*RPPA, reverse phase microarray; response was detected according to International Working Group (IWG) response criteria*.

All patients included in the study provided written informed consent, in accord to Declaration of Helsinki.

### Protein detection using reverse phase micro array (RPPA)

Protein amount in each sample was detected by RPPA, as previously described (Mueller et al., 2010). Briefly, samples were washed in PBS twice to remove the fixative, lysed in 40 uL protein extraction buffer (equal volumes of 2-Tris-Glycine SDS Sample Buffer (Invitrogen, Carlsbad, CA), and T-PER Tissue Protein Extraction Reagent (Pierce/Thermo-Fisher, Rockford, IL) plus 1.0% 2-β-mercaptoethanol (Sigma-Aldrich) at an approximate ratio of 1,000 cells/μL. Proteins were denatured by heating for 5 min at 100 C prior to dilution in the microtiter plate. Serial 2-fold dilutions of the lysates were printed in duplicate on glass backed nitrocellulose array slides (Nexterion Slides, Schott, Elmsford, N) in a dilution curve representing undiluted lysate and 1:2, 1:4, and negative control dilutions, using an Aushon 2470 arrayer (AushonBiosystems, Billerica, MA) equipped with 350 μM pins. Each spot was printed with approximately 30.0 nL of lysate/spot. The slides were stored with desiccant (Drierite, W. A. Hammond, Xenia, OH) at −20°C prior to immunostaining.

Printed slides were prepared for staining by treating with 1xReBlot (Millipore, Billerica, MA) for 15 min, followed by 2 × 5 min washes with PBS. Slides were treated for 1 h with blocking solution (1 g of I-block (Applied Biosystems, Bedford, MA), 0.5% Tween-20 in 500 mL of PBS) with constant rocking at room temperature. Each slide was incubated with a single primary antibody at room temperature for 30 min.

Each array was probed with a single polyclonal or monoclonal primary antibody.

Primary antibodies used to investigate 25 endpoints for RPPA are listed in Table 2. Each antibody was subjected to validation by immunoblotting prior to use on the RPPA. Antibody validation criteria included detection of a single band at the appropriate molecular weight in positive control lysates and the absence or significantly reduced presence of a band in negative control lysates by immunoblotting.

**Table 2:** **List of endpoints and dilution of antibodies used on RPPA**.

	**Manifacturer**	**Cat #**	**Dilution for arrays**
Akt Ser473	CellSig	9,271	1:100
Akt Thr308	CellSig	9,271	1:100
Atg5 (part of Autophagy Ab Sampler #4445)	CellSig	2,630	1:500
Beclin-1 (part of Autophagy Ab Sampler #4445)	CellSig	3,738	1:500
c-Abl Thr735	CellSig	2,864	1:1,000
CHK1 Ser345	CellSig	2,341	1:100
c-myc	CellSig	9,402	1:200
Cofilin (S3) (77G2)	CellSig	3,313	1:1,000
ERK 1/2 Thr202/Tyr204	CellSig	9,101	1:1,000
Ezrin Tyr353	CellSig	3,144	1:1,000
Foxo1A/3A	NovusBiol	NBP1-70789	1:1,000
HDAC1	CellSig	2,062	1:100
HDAC3	CellSig	2,632	1:1,000
LC3B (part of Autophagy Ab Sampler #4445)	CellSig	2,775	1:500
mTOR Ser2448	CellSig	2,971	1:1,000
Musashi	CellSig	2,154	1:500
Notch 1	Millipore	AB5707	1:1,000
NUMB	Abcam	ab14140	1:500
p53	CellSig	9,282	1:1,000
phospho-p53 Ser15	CellSig	9,283	1:1,000
PLCY1 Tyr783	CellSig	2,821	1:1,000
Shc Tyr317	Upstate	07-206	1:1,000
Src Tyr416	CellSig	2,101	1:250
STAT3 Tyr705	CellSig	9,145	1:400
STAT5 Tyr694	CellSig	9,351	1:1,000

The negative control slide was incubated with antibody diluent. Secondary antibody was goat anti-rabbit IgG heavy + chain (1:10,000) (Vector Laboratories, Burlingame, CA). Subsequent protein detection was amplified via horseradish peroxidase-mediated biotinyltyramide with chromogenic detection (diaminobenzidine) according to the manufacturer's instructions (Dako).

Total amount per microarray spot was normalized on total DNA level as previously described (Chiechi et al., 2012, 2013), and confirmed on total protein and housekeeping protein beta-actin. Total protein staining was performed using Sypro Ruby Protein blot stain (Invitrogen) according to the manufacturer's instructions and scanned with a NovaRay CCD imager (Alpha Innotech, San Leonardo, CA, USA) equipped with a Cy3 filter.

The stability of three normalization analytes (ssDNA, total protein and β-actin) and three protein analytes (Akt Ser473, Akt Thr308, ERK Thr202/Tyr204) was evaluated by geNorm and NormFinder as previously reported (Chiechi et al., 2012).

Arrays were scanned, spot intensity analyzed with commercial software Image Quant v.5.0, data normalized, and a standardized, single data value was generated for each sample on the array. Additional quality control measures for antibody staining included evaluation of reference lysate staining by visual inspection of scanned images and examination of data analysis results for the positive and negative reference lysates.

### Cell culture

Primary samples were processed upon obtainment of patient's informed consent, in accordance with the Declaration of Helsinki. Bone marrow mononuclear cells (BMMCs) were purified by standard density gradient centrifugation (Ficoll-Hypaque, GE Healthcare, Roosendal, The Netherlands) and cultured in RPMI medium (Gibco-Life Technologies, 31860) supplemented with 10% fetal bovine serum (FBS), penicillin (100 U/mL), and streptomycin (100 μg/mL) at 37°C in a humidified atmosphere containing 5% CO_2_.

Cell counts were performed with Countess Automated Cell Counter (Invitrogen, Life Technologies). Briefly, 20 μl cell suspensions were mixed with 20 μl 0.4% Trypan Blue solution and loaded into Countess cell counting chamber slides. The protocol was customized based on sensitivity, maximum and minimum size and accurate cell density range (2 × 104 to 2.5 × 106 cells/ml).

Viability of cells was also evaluated by the ATP-lite1step assay (PerkinElmer, Monza, Italy), as described by the manufacturer. Briefly, cells were plated onto 96-wells microplates in 100 uL growth medium and 100 uL of the reconstituted reagent was added to each well. Cells were incubated at 37°C for 20 min in the dark. Luminescence was measured using a Victor3 (PerkinElmer).

### Drug treatments

Azacitidine (5-AZA, kindly given by Celgene Italia), Chloroquine (CQ, Sigma, St. Louis, MO) and Leupeptin (Leu, Sigma, St. Louis, MO) were freshly prepared in RPMI1640 medium for each experiment.

### Cell cycle analysis

After 24, 48, and 72 h, cells were washed and resuspended in cold 80% ethanol to a final concentration of 0.5^*^10^∧^6 cells/mL for 1 h at 4C. The ethanol-fixed cells were centrifuged to remove ethanol and the pellet was resuspended in propidium iodide-staining reagent (0.1% triton X-100, 0.1 mm EDTA, 0.05 mg/mLRNase A and 50 lg/mL propidium iodide). Cells were stored in the dark at room temperature for 3 h. Cells were then analyzed with a flow cytometer (FC500 Beckman coulter; Beckman Coulter S.p.A., Milano, Italy) and processed by Mod Fit program.

### Immunoblot analysis

Cells were lysed for 10 min on ice in TBS buffer (150 mM NaCl, 10 mM Tris-HCl, pH 7.5) and 1% SDS supplemented with protease inhibitors. Genomic DNA was sheared by 10 min sonication. After lysis, proteins were quantified by Bradford assay (Bio-Rad Protein assay, Bio-Rad Laboratoires GmbH). 30 μg proteins were resolved by 8-10-15% SDS-PAGE and blotted on nitrocellulose (Mini-PROTEAN Tetra Cell, BioRad). Membranes were blocked with 5% milk in TBS buffer, incubated with primary and secondary antibodies diluted in 5% BSA in TBS buffer, washed with 0.1% TBS-Tween, and proteins revealed by ECL (SuperSignal West Pico Chemiluminescent substrate, Thermo Scientific) at ChemiDoc-it (UVP) for HRP-conjugated secondary antibody. The following antibodies were used at the indicated dilutions: rabbit anti-p62 (1:1,000; Sigma-Aldrich Co.); mouse anti-beta-actin (1:3,000; A5441, Sigma-Aldrich Co.).

### Statistical methods

Mann-Whitney test was used to compare values between two groups. *p* < 0.05 were considered statistically significant. All calculations were performed using GraphPad Prism version 6.00 for Windows, GraphPad Software, San Diego California USA, www.graphpad.com.

## Results

### Pro-survival pathways are elicited by long-term exposure to 5-AZA

To investigate survival pathways elicited by long-term exposure to 5-AZA *in vivo* BMMCs were collected from newly-diagnosed MDS patients (Table 1) before (T0) and after first four 5-AZA cycles (T1).

**Figure 1:**
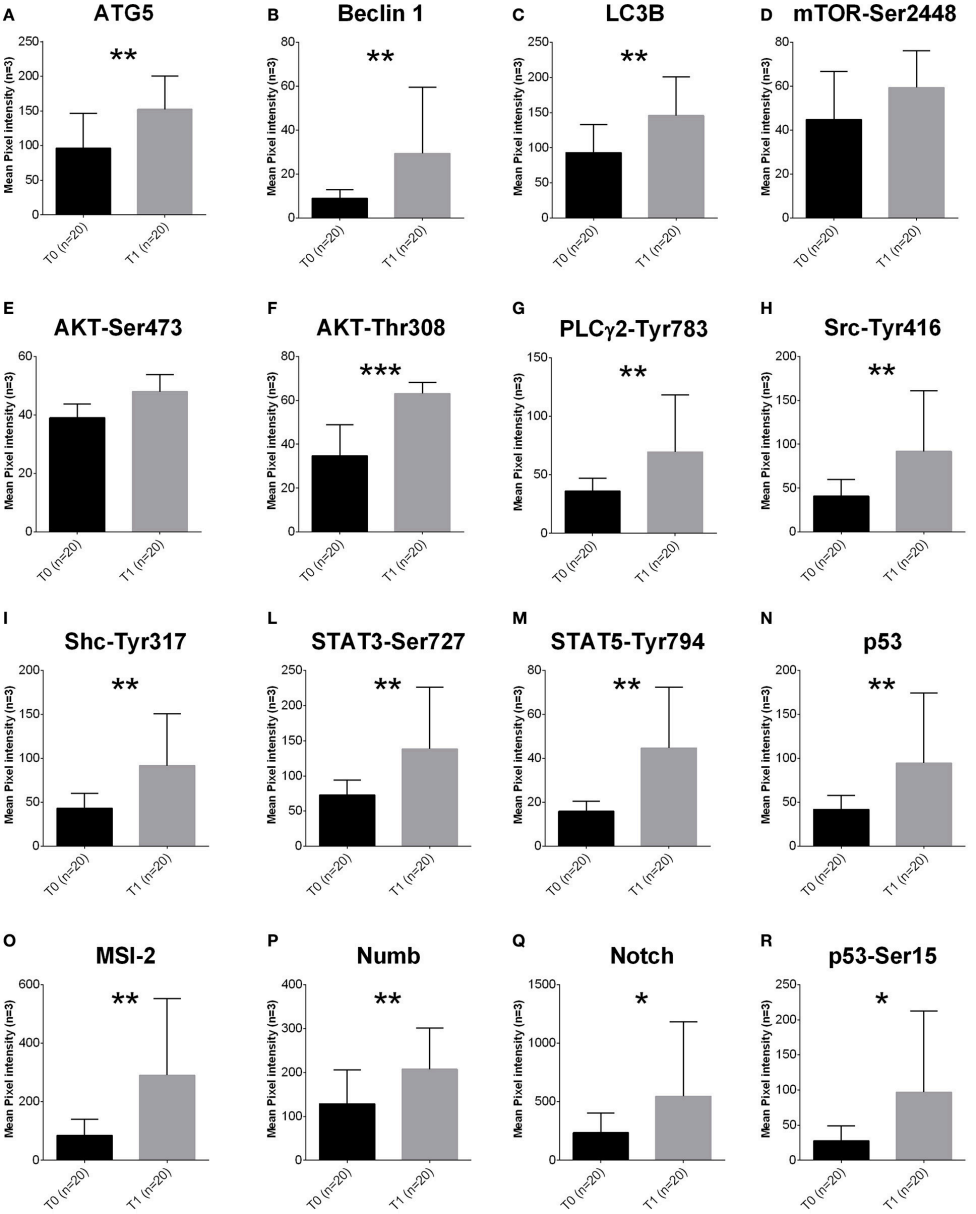
**Proteomic profile of MDS-BMMCs after long-term exposure to 5-AZA ***in vivo*****. **(A–R)** Proteins obtained from BMMCs isolated from patients at diagnosis (gray bars) and after treatment (black bars) with 4 cycles of 5-AZA were analyzed using RPPA to as described in the Materials and Methods section. Results represent the mean of 20 observations in triplicate; error bars denote standard deviation. ^*^p < 0.05, ^**^p < 0.001, ^***^p < 0.0001 (Mann-Whitney test). The label indicates the endpoint tested (**A–C** autophagy pathway, **D–F** proliferation, **G–R** intracellular signaling).

To quantify pro-survival signaling we investigated the amount of proteins involved in the autophagy pathway. ATG5 (*p* < 0.0001), Beclin 1 (*p* = 0.0056) and LC3B (*p* = 0.0124) were increased in T1 samples (Figures 1A–C).

Activation of the autophagy pathway occurred independently from activation of mTOR, since mTORSer2448 (Figure 1D), AktSer473 (Figure 1E) and ERKThr202Tyr204 were not affected (Supplementary Figure 1). However, increased levels of AktThr308 (Figure 1F) were associated to increased PLC-Y-Tyr783, and its targets upstream SrcTyr416, ShcTyr317, and downstream and STAT3-Ser727 and STAT5-Tyr694 (Figures 1I–M).

Survival signaling was associated to increased expression of Musashi (MSI-2), a key player of progression from MDS to AML (Kharas et al., 2010) and its downstream targets Numb, Notch and active p53 (p53Ser15, Figures 1N–R).

In order to exclude any potential artifact, we included an additional normalization to total proteins and beta-actin confirming the results described above (Supplementary Figures 2A,B).

### Targeting autophagy improve cytotoxic effect of 5-AZA

**Figure 2:**
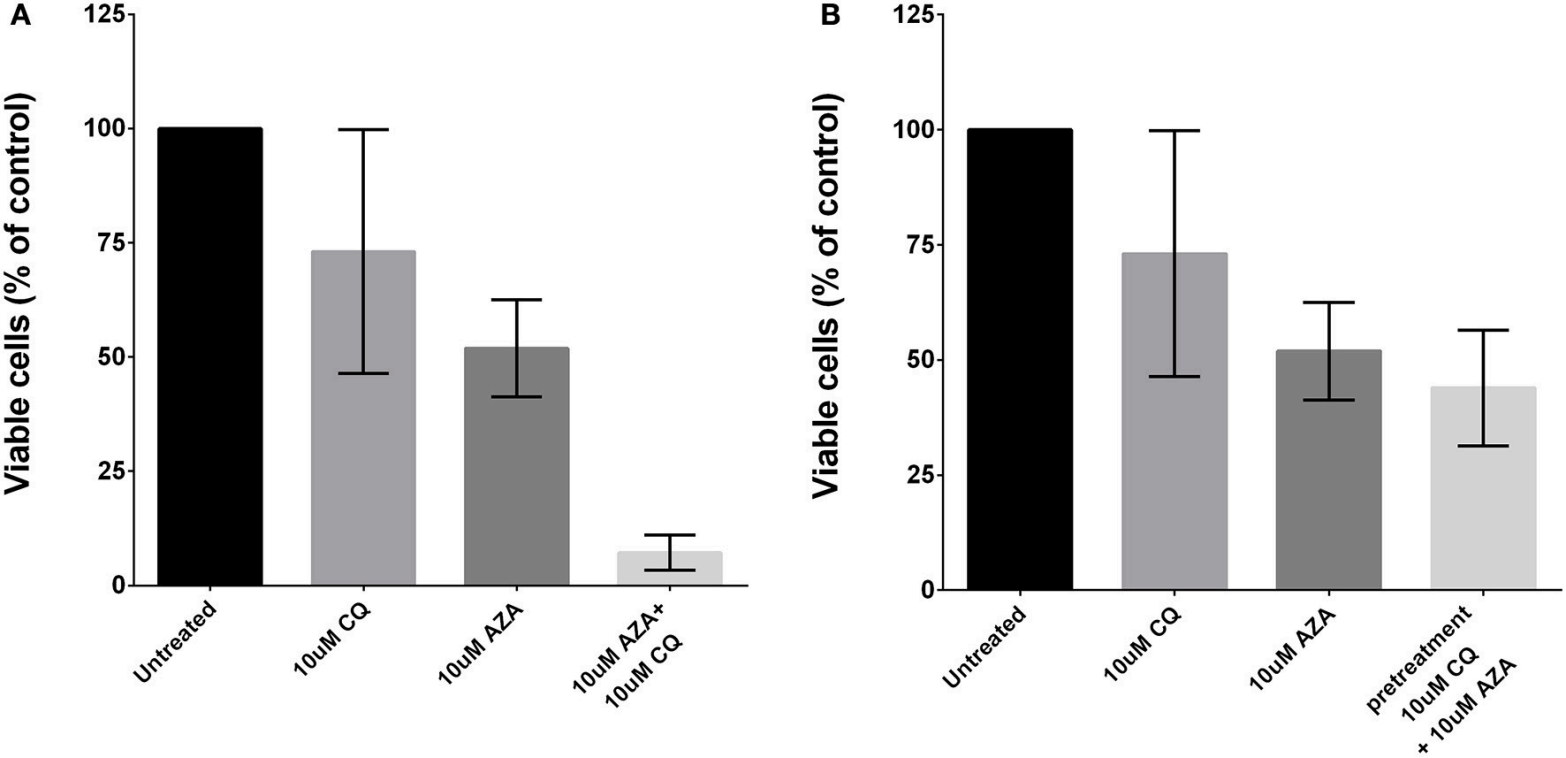
**Effects of autophagy inhibition and 5-AZA treatment in MDS-BMMCs ***in vitro*****. Cell viability was assessed after 72 h of treatment with AZA or CQ or their combination (10 μM) using ATP-lite assay, showing a synergic effect **(A)**. Pre-treatment with CQ (10 μM) for 24 h and addition of 10 μM AZA for 72 h did not exhibit a synergic effect of the two drugs **(B)**. Standard deviation was determined from 8 independent experiments, including triplicate wells per experiment.

To assess whether autophagy contributes to 5-AZA sensitivity escape of MDS cells, we treated primary MDS BMMCs (*N* = 8) with a sub-lethal dose of 5-AZA (5 μM) and with the prototypical lysosomotropic autophagy inhibitor chloroquine (CQ), alone or in combination for 72 h, and found these treatments to exert significant synergistic toxicity (Figure 2A).

Since mice lacking Atg7 in HSCs develop an atypical myeloproliferation resembling human MDS progressing to AML as consequence of accumulation of damaged mitochondria and reactive oxygen species (ROS) (Mortensen et al., 2011; Watson et al., 2011), we first investigated the effects of 5-AZA after basal autophagy inhibition.

Thus, we tested the effect of pre-treatment with 10 μM CQ for 24 h followed by treatment with 5 μM 5-AZA for 72 h, and found this combination to exert a modest decrease of viability (Figure 2B).

### Inhibiting autophagy after long-term exposure to 5-AZA improves cytotoxic effect

**Figure 3:**
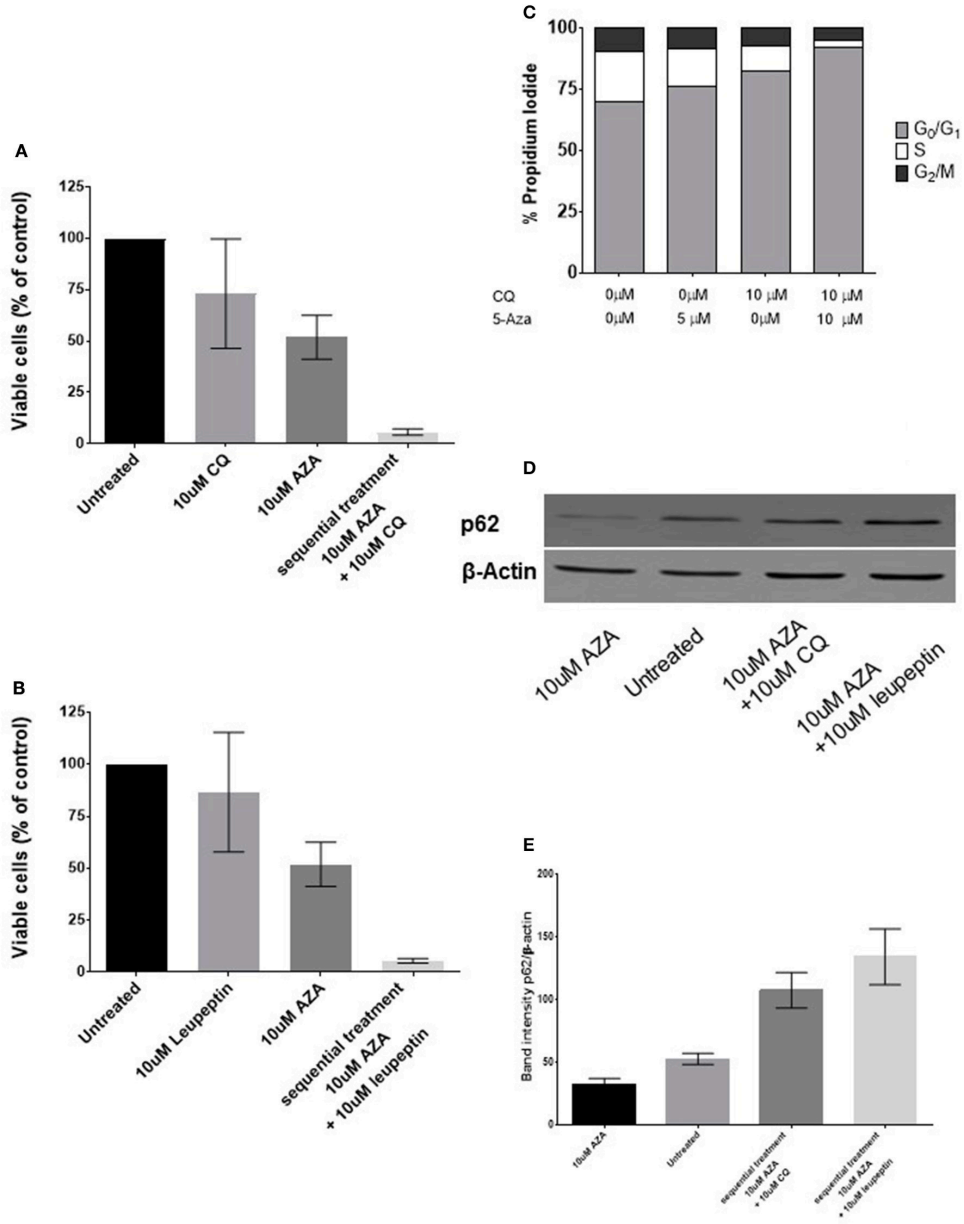
**Effects of sequential treatment with 5-AZA followed by CQ on MDS-BMMCs ***in vitro*****. BMMCs were treated with 5 μM 5-AZA for 72 h, washed in PBS and exposed to 10 μM CQ **(A)**, 10 μM leupeptin for further 24 h **(B)**. When cells were treated with CQ after 72 h of treatment with 5-AZA, a change in cell cycle was appreciated **(C)**. The effect on the autophagic marker SQSTM1/p62 after 72 h of treatment with 5-AZA followed or not by 10 μM CQ or leupeptin was evaluated by WB **(D**,**E)**. Standard deviation was determined from 6 independent experiments, including triplicate wells per experiment.

Since previous findings suggested that synergism was due to an increase of basal autophagy due to 5-AZA exposure, we adopted a sequential treatment treating BMMCs with 5 μM 5-AZA for 72 h followed by 10 μM CQ for 24 h. We found that 5-AZA lethality was increased using the sequential approach with CQ (*p* = 0.003, Figure 3A) or leupeptin (*p* = 0.003, Figure 3B), associated to a reduction of G2M phase and increase in G0-G1 phase (*p* = 0.0056, Figure 3C).

Since CQ inhibits fusion of autophagosomes with lysosomes without being a specific autophagy inihibitor, we used the same approach treating 72-h 5-AZA exposed BMMCs with the distal autophagy inhibitor leupeptin as well and confirmed its synergic effect with 5-AZA (Figure 3B).

Finally, we measured autophagy biochemically, under basal condition and after treatment with 5-AZA for 72 h followed by treatment with CQ or leupeptin, using the autophagic receptor SQSTM1/p62. The decrease of p62 upon 5-AZA exposure alone suggests an autophagic induction by 5-AZA, reversible by CQ or leupeptin exposure (Figures 3C,D).

## Discussion

Clinical studies have been shown the 5-AZA efficacy in high-risk MDS patients. The beneficial effects were noted after approximately four cycles and continued up to eight cycles (Santini et al., 2010; Silverman et al., 2011). 5-AZA delayed the onset of acute myeloid leukemia (AML) but after drug interruption the progression to AML was inevitable (Fenaux et al., 2009; Seymour et al., 2010; Silverman et al., 2011; Zeidan et al., 2014).

Several strategies have been evaluated after failure of treatment with 5-AZA, included rigosertib (Garcia-Manero et al., 2016), decitabine (Harel et al., 2015) and vorinostat (Prebet et al., 2014), with dismal outcome. Thus, understanding pro-survival pathways elicited by long-term exposure to 5-AZA is a clinical need for tailored salvage therapy.

The molecular basis for 5-AZA efficacy is unclear, whether due to induction of apoptosis and how MDS clones can displace progressively the normal hematopoiesis or to restoring gene expression and blast differentiation, reducing methylation of aberrant silencing of key genes (Tefferi and Vardiman, 2009; Buckstein et al., 2011).

Potential target proteins include those of p53 family, affecting cell differentiation and apoptosis, or the p21 and p18 affecting the behavior of stem cells, and altered p38 mitogen-activated protein kinase (MAPK) activation (Navas et al., 2006). Little is known about pro-survival compensatory pathways induced by chronic exposure to 5-AZA. In our work, we investigated the role of autophagy in MDS patients who achieved at least a hematological response after the first four 5-AZA cycles, in order to evaluate molecular markers in the window of clinical benefit.

The characterization of biologic properties of myelodysplastic cells *in vitro* is hampered by a lack of animal models (Komeno et al., 2009) and by difficulties in isolating the cell populations responsible for the disease and/or its maintenance, thus some speculations are only possible based on clinical observations. Our data are not able to show the differentiation pathway from the neoplastic proliferation itself, since we could not separate neoplastic from non-neoplastic cells due to a lack of unique surface antigens on the BMMCs milieu.

BMMCs of patients with MDS have altered signal transduction pathways. For example, the erythropoietin receptor is expressed at a normal density on MDS cells, but STAT5 activation in response to erythropoietin stimulation is defective (Mittelman et al., 1996; Hoefsloot et al., 1997; Shimizu et al., 1999). Few studies have been conducted regarding the basal activation of proliferative signaling in MDS marrow progenitors (Hoefsloot et al., 1997; Fontenay-Roupie et al., 1999; Spinelli et al., 2012), and none after exposure to drugs.

Autophagy signaling is needed for survival and proliferation in stressful conditions, and it is emerging as a novel pathway to modulate in progression from MDS to AML (Watson et al., 2011). Our findings overlap with emerging data in the field which has been recently published (Follo et al., 2008, 2009, 2011, 2012). Mice with autophagic defects develop an atypical myeloproliferation resembling MDS progressing to AML (Mortensen et al., 2011); while in our *in vitro* experiments, increased expression of proteins involved in the autophagy pathway (ATG5, Beclin and LC3B) was connected to long-term exposure to 5-AZA. After a median follow-up of 21 months all patients but one progressed to AML and further analyses are ongoing to explore the role of autophagy in progression to AML.

Despite several reports in the field, the role of autophagy in MDS is not clear, and could have an opposite role in patients with low or high-risk disease. Indeed, in high-risk disease autophagy could be implicated in the resistance to apoptosis in MDS progenitors. In low-risk MDS, autophagy in erythroid cells has been shown to enhance the physiological clearance of mitochondria during terminal differentiation, thus removing defective iron-laden mitochondria (Houwerzijl et al., 2009).

Our proteomic approach revealed that 5-AZA long-term treatment induces autophagy in MDS-BMMCs, through several triggers: (i) increase of active Abl-Tyr735; (ii) increase of PLC-γ-Tyr783 which activates AKT (Wang et al., 2006); (iii) increase of p62, associated with phosphorylation of AKT-Thr308. Our study confirms in the MDS setting that RPPA technology is suitable for drug and biomarker discovery as a result of the high-sensitivity, high-reproducibility, high-throughput, and quantitative features of the approach. Recent studies have applied RPPA to determine the mechanism of action and selectivity of emerging drug candidates at the pathway level, as well as to uncover unexpected drug resistance mechanisms.

Abl and its effector Src has been reported as regulator of late stages of autophagy. Defective lysosomal degradation of long-lived proteins in the absence of Abl kinase signaling was accompanied by a perinuclear redistribution of lysosomes and increased glycosylation and stability of lysosome-associated membrane proteins, which are known to be substrates for lysosomal enzymes and play a role in regulating lysosome mobility (Yogalingam and Pendergast, 2008).

Recent reports showed that PI-PLCβ1 was associated with activated Akt levels in high-risk MDS (Follo et al., 2008) and that a specific increase of PI-PLCβ1 mRNA within the first 3 cycles of 5-AZA correlated with a longer duration of response and with an increase in myeloid differentiation (Cocco et al., 2015). Our study also reveals that PLC-γ is associated with increased AKT, and could contribute in the same way as PI-PLCβ1 in mediating 5-AZA responsiveness.

Activation of AKT through phosphorylation of Threonine308 has recently been found to be associated with increased levels of p62 during the initial induction of p62. The translocation of Akt-Phafin2 in the nucleus to the lysosome is essential for autophagy activation (Matsuda-Lennikov et al., 2014).

After 5-AZA exposure, autophagy flux was increased as suggested by increase of ATG5, Beclin-1 and LC3-B (Figures 1A–C) and p62 reduction (Figures 3D,E). When we added chloroquine or leupeptin, authophagy flux was inhibited, p62 could not be degraded and accumulated in the cells (Figures 3D,E). Inhibiting the fusion of autophagosomes with lysosomes with chloroquine increased cytotoxic effect of 5-AZA (Figure 3B). On the opposite, leupeptin, a distal autophagy inhibitor increased cytotoxic effect of 5-AZA associated to p62 accumulation in the cells.

Chloroquine is a lysosomotropic agent that prevents endosomal acidification and is commonly used to study the role of endosomal acidification in cellular processes and inhibit early phases of autophagy *in vitro* and *in vivo* (Greenberg et al., 1997; Sanz et al., 1998; Amaravadi et al., 2007; Carew et al., 2007; Maclean et al., 2008; Bellodi et al., 2009; Milan et al., 2015). Chloroquine is used therapeutically in the context of clinical trials due to its manageable toxicity profile as recently reviewed (Rebecca and Amaravadi, 2016).

Finally, 5-AZA long-term exposure was associated to progression in cell-cycle, with an increase of transcriptional factors based on our proteomics screening: (i) Msi-2; (ii) c-myc; (iii) STAT-3, and STAT-5.

Previous work showed that Msi-2 overexpression and knockdown strategies influence proliferation and differentiation of HSCs and myeloid progenitors, through increased c-myc levels, associated with blastic crisis in chronic myeloid leukemia and poor outcome in AML (Kharas et al., 2010). The hypothesis that Msi-2 increases after 5-AZA long-term exposure could be involved in the progression from high-risk MDS through AML is currently under investigation in our laboratory.

## Conclusions

Based on our data, prolonged exposure to 5-AZA can induce pro-survival signaling, including autophagy, without affecting proliferation. We suggest that 5-AZA long-term treatment can favor compensatory pro-survival pathways that can emerge when the drug exposure is interrupted and confer advantage to malignant clones. Targeting autophagy after 5-AZA wash-out could delay the emergence of this potential malignant signaling.

## Ethics statement

This study was carried out in accordance with the recommendations of “Comitato Etico AOUP Policlinico di Catania” with written informed consent from all subjects. All subjects gave written informed consent in accordance with the Declaration of Helsinki. The protocol was approved by the “Comitato Etico AOUP Policlinico di Catania,” protocol EMADRMA number 34/2013/VE.

## Author contributions

AR, PL, and NP provided samples and performed experiments; AC, LL, CV, VE, and AR performed RPPA and proteomic data analysis; CG and DT analyzed results; AR, AC, and NP analyzed results and made the figures; AR, GP, and FD designed the research and wrote the paper.

## Funding

This work was supported in part by a grant from Celgene, by Associazione Italiana contro le Leucemie (AIL) of Catania and Fondazione Catanese per lo Studio e la Cura delle Malattie Neoplastiche del Sangue (FON.CA.NE.SA) and Ministero della Salute (Ricerca Finalizzata PE-2011-02350147).

### Conflict of interest statement

FD and AR received honoraria and grants by Celgene. The other authors declare that the research was conducted in the absence of any commercial or financial relationships that could be construed as a potential conflict of interest.

## Supplementary material

Supplementary Figure 1 **Survival signaling pathway investigated by RPPA in MDS-BMMCs (A)**. Proteins obtained from BMMCs isolated from patients at diagnosis (gray bars) and after treatment (black bars) with 4 cycles of 5-AZA were analyzed using RPPA as described in the Methods section. Results represent the mean of 20 observations in triplicate; error bars denote SEM. Stars report *p* < 0.005 (Mann-Whitney test). 5-AZA = 5-azacitidine **(B)**. The array for detection of ATG5 by RPPA is shown as example of RPPAs.

Supplementary Figure 2 **Survival signaling pathway investigated by RPPA in MDS-BMMCs**. Proteins obtained from BMMCs isolated from patients at diagnosis (gray bars) and after treatment (red bars) with 4 cycles of 5-AZA were analyzed using RPPA as described in the Methods section. Results represent the mean of 20 observations in triplicate, normalized on the amount of total protein **(A)** or Beta-Actin **(B)**; error bars denote SEM. Stars report *p* < 0.005 (Mann-Whitney test). 5-AZA = 5-azacitidine. The effect on the autophagic marker SQSTM1/p62 after 72 h of treatment with 5-AZA was evaluated by WB.
