# Supplementary figures and images for: Proteomic Analysis Reveals Autophagy as Pro-Survival Pathway Elicited by Long-Term Exposure with 5-Azacitidine in High-Risk Myelodysplasia
Source: Front Pharmacol. 2017 Apr 26;8:204. doi: 10.3389/fphar.2017.00204 (PMC5405131; doi:10.3389/fphar.2017.00204)

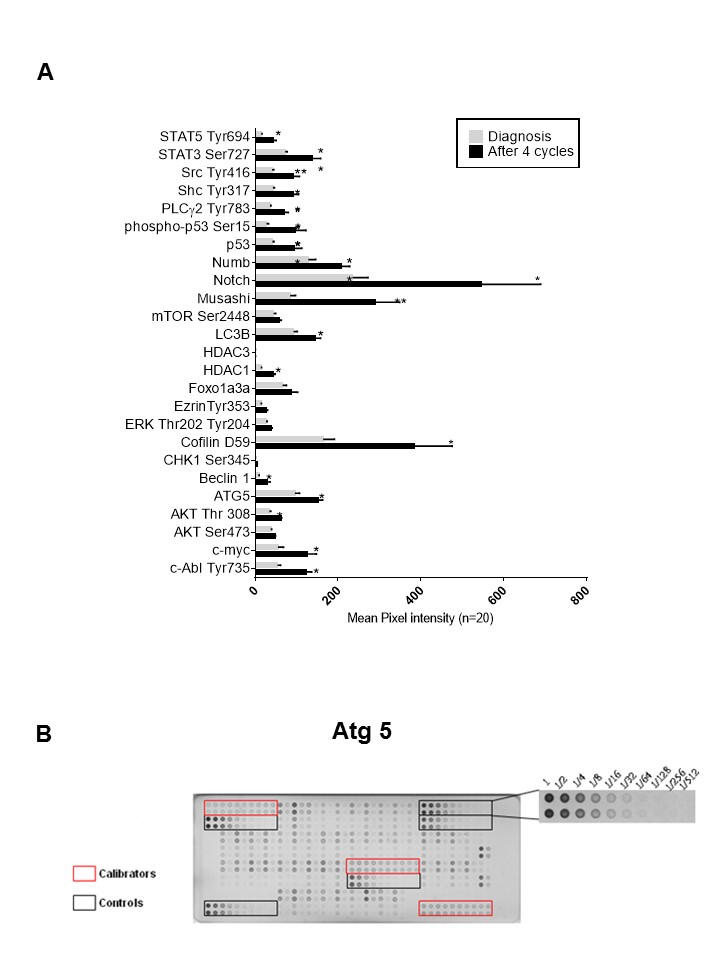

Supplement: Supplementary Figure 1 — Survival signaling pathway investigated by RPPA in MDS-BMMCs (A). Proteins obtained from BMMCs isolated from patients at diagnosis (gray bars) and after treatment (black bars) with 4 cycles of 5-AZA were analyzed using RPPA as described in the Methods section. Results represent the mean of 20 observations in triplicate; error bars denote SEM. Stars report p < 0.005 (Mann-Whitney test). 5-AZA = 5-azacitidine (B). The array for detection of ATG5 by RPPA is shown as example of RPPAs. [file Image1.JPEG]

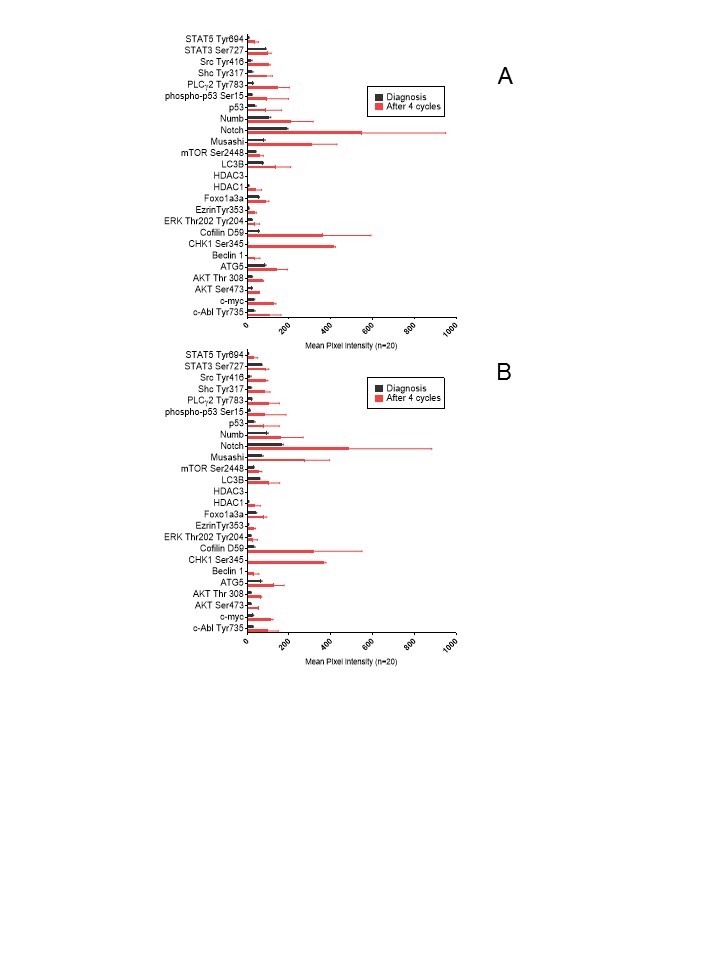

Supplement: Supplementary Figure 2 — Survival signaling pathway investigated by RPPA in MDS-BMMCs. Proteins obtained from BMMCs isolated from patients at diagnosis (gray bars) and after treatment (red bars) with 4 cycles of 5-AZA were analyzed using RPPA as described in the Methods section. Results represent the mean of 20 observations in triplicate, normalized on the amount of total protein (A) or Beta-Actin (B); error bars denote SEM. Stars report p < 0.005 (Mann-Whitney test). 5-AZA = 5-azacitidine. The effect on the autophagic marker SQSTM1/p62 after 72 h of treatment with 5-AZA was evaluated by WB. [file Image2.JPEG]
